# Reference Values of the Quality of Life after Brain Injury (QOLIBRI) from a General Population Sample in Italy

**DOI:** 10.3390/jcm12020491

**Published:** 2023-01-06

**Authors:** Ugne Krenz, Sven Greving, Marina Zeldovich, Juanita Haagsma, Suzanne Polinder, Nicole von Steinbüchel, on behalf of the CENTER-TBI Participants and Investigators

**Affiliations:** 1 Institute of Medical Psychology and Medical Sociology, University Medical Center Göttingen, Waldweg 37A, 37073 Göttingen, Germany; 2 Department of Public Health, Erasmus MC, University Medical Center Rotterdam, 3000 CA Rotterdam, The Netherlands

**Keywords:** QOLIBRI, TBI-specific HRQoL instrument, health-related quality of life, traumatic brain injury, reference values, healthy individuals, chronic health condition

## Abstract

Traumatic brain injury (TBI) may affect the lives of the individuals concerned and their relatives negatively in many dimensions. Health-related quality of life (HRQoL) is a comprehensive and complex concept that can assess one’s satisfaction with a broad range of areas of life and health. The Quality of Life after Traumatic Brain Injury (QOLIBRI) questionnaire is a TBI-specific measure for HRQoL which is used in research and health services worldwide. When evaluating self-reported HRQoL after TBI, reference values from a general population are helpful to perform clinically relevant evaluations and decisions about the condition of an affected person by comparing the patient scores with reference values. Despite the widespread use of the QOLIBRI, reference values have until now only been available for the Netherlands and the United Kingdom. The aim of this study was to validate the QOLIBRI for the general population in Italy and to provide reference values. An adapted form of the QOLIBRI was administered to 3298 Italians from a healthy general population using an online survey. Their scores were compared with those of 298 individuals post-TBI recruited within the international longitudinal observational cohort CENTER-TBI study in Italian hospitals, who completed the original questionnaire. The psychometric characteristics and the measurement invariance of the QOLIBRI were assessed. A regression analysis was performed to identify predictors relevant for HRQoL in the general population. Reference values were provided using percentiles. Measurement invariance analysis showed that the QOLIBRI captures the same HRQoL constructs in an Italian general population and Italian TBI sample from the observational Center-TBI study. Higher age, higher education and the absence of a chronic health condition were associated with higher QOLIBRI scores, suggesting better HRQoL. Reference values were provided for a general Italian population adjusted for age, sex, education and presence of chronic health conditions. We recommend using these for a better interpretation of the QOLIBRI score in clinical practice and research in Italy.

## 1. Introduction

Traumatic brain injury (TBI) is an important cause of burden of disease worldwide, as more than 50 million people acquire it every year [1]. In a study published in 2018, Dewan et al. [2] estimated that approximately 69 million people worldwide experience TBI each year. In Italy, the incidence of TBI varies from 212.4 [3] to 848 [4,5] cases per 100,000, depending on the study, placing it among the countries with the highest TBI rates in Europe.

TBI negatively affects the lives of individuals after TBI and their relatives [6,7] by limiting their everyday lives, causing physical [8], cognitive [9] and psychological problems [10], and having negative effects on their emotions [11] and social lives [12,13]. Because of the long-term outcomes, which are similar to those caused by a chronic health condition, TBI has been equated with chronic diseases [14]. In recent decades, the description and treatment of chronic diseases has seen a shift from a biomedical to a biopsychosocial approach to disease and health. Consequently, health is seen as a multidimensional construct that includes physical and psychosocial aspects [15]. In their systematic review, Polinder et al. [16] point out that TBI has a relevant impact on the health-related quality of life (HRQoL) of the individuals concerned.

HRQoL is a comprehensive and complex construct which includes a broad range of areas of life and health. It covers self-reported outcomes on health status and well-being, and can be used to determine the effectiveness of a treatment [16,17]. Sherer and his colleagues [18] postulated that physical function, physical symptoms, cognition, negative and positive emotions, sense of self, and social participation provide a differentiated foundation for understanding the HRQoL of individuals after TBI. HRQoL can be assessed using disease-specific or generic instruments. Generic instruments can be used in the assessment of HRQoL after TBI [19], especially when comparisons are to be made with other diseases [20]. However, these instruments are described as being less sensitive to specific health conditions, which is why the use of disease-specific instruments is recommended [21,22]. Specific instruments are generally more sensitive and more responsive [23] to the problems of a particular disease area and can capture HRQoL more precisely [24]. For example, Harfmann et al. [19] have compared specific and generic instruments in patients after TBI and shown that the TBI-specific measures offer a more detailed assessment of symptoms relevant to TBI than generic ones.

The instrument measuring Quality of Life after Traumatic Brain Injury (QOLIBRI) is the first disease-specific questionnaire that captures HRQoL after TBI [25,26]. It covers all aspects suggested by Sherer et al. [18] within 37 items forming six subscales (cognition, self, autonomy and daily life, social, emotions, physical). The QOLIBRI helps to identify self-perceived deficits that should be further investigated and, if possible, improved. This instrument was developed in 2010 and was validated for the Italian language in 2014 [27], showing good psychometric characteristics.

The QOLIBRI instrument is applied in various settings in the area of TBI, from international research studies to clinical use [28,29,30] and rehabilitation [31]. Until now the QOLIBRI has been translated and validated in more than 26 languages and is widely used internationally for individuals after TBI [31,32,33,34]. However, to better understand the clinical impact of TBI on the HRQoL of patients, reference values for individuals from comparable general populations are required. Reference values are important, e.g., in order to evaluate the HRQoL of an individual after TBI in relation to a comparable general population, so as to capture the HRQoL domains showing deficits. To date, no reference values exist for the Italian version of the QOLIBRI.

Since the QOLIBRI is a TBI-specific measure, it should be adapted for use in the general population. To ensure comparability of the QOLIBRI scores between individuals after TBI and the general population, evidence of measurement invariance (MI) is crucial. MI in this sense means that any observable variation in (adapted) QOLIBRI responses between TBI and the general population can be attributed to real differences in HRQoL. The aim of this study was therefore to validate the QOLIBRI questionnaire for a sample from the Italian general population in order to compile reference values and to compare these with the QOLIBRI scores of individuals after TBI.

## 2. Methods

### 2.1. Study Design

This study includes data from two different sources. Data for the general population sample from Italy are derived from a web-based, self-reported, cross-sectional study. The data for individuals after TBI stem from the multicenter, prospective, longitudinal, observational Collaborative European Neuro Trauma Effectiveness Research in Traumatic Brain Injury study (CENTER-TBI; clinicaltrials.gov NCT02210221). For details on enrollment of participants and recruitment, see Steyerberg et al. [35].

### 2.2. Ethical Approvals

#### 2.2.1. General Population Sample

The study on general population was a part of the CENTER-TBI project. Ethical approval was obtained from the Leids Universitair Centrum—Commissie Medische Ethiek (approval P14.222/NV/nv, 3 December 2014).

#### 2.2.2. TBI Sample

The CENTER-TBI study (EC grant 602150) has been conducted in accordance with all relevant laws of the EU if directly applicable or of direct effect and all relevant laws of the country where the recruiting sites were located, including, but not limited to, the relevant privacy and data protection laws and regulations (the “Privacy Law”), the relevant laws and regulations on the use of human materials, and all relevant guidance relating to clinical studies from time to time in force, including, but not limited to, the ICH Harmonized Tripartite Guideline for Good Clinical Practice (CPMP/ICH/135/95) (“ICH GCP”) and the World Medical Association Declaration of Helsinki entitled “Ethical Principles for Medical Research Involving Human Subjects”. Informed consent was obtained for all patients recruited in the Core Dataset of CENTER-TBI and documented in the e-CRF. Ethical approval was obtained for each recruiting site. The list of sites, Ethical Committees, approval numbers and approval dates can be found on the project’s website https://www.center-tbi.eu/project/ethical-approval (accessed on 15 July 2022).

### 2.3. Instruments

#### 2.3.1. Quality of Life after Traumatic Brain Injury (QOLIBRI)

The QOLIBRI is the first instrument specifically developed for individuals after TBI to assess their disease-specific HRQoL. It comprises 37 items associated with four scales (Cognition, Self, Daily Life and Autonomy, and Social Relationships) with items measuring satisfaction with various aspects of HRQoL (part A) and two scales (Emotions and Physical Problems) measuring issues that individuals after TBI feel bothered by (part B). Responses to the Part A items are coded on a 5-point Likert scale with 1 corresponding to not at all satisfied and 5 to very satisfied. Responses to the items in Part B are reversely scored to correspond with the items of the Part A. Here, 1 indicates very (bothered) and 5 means not at all bothered. Like other instruments measuring quality of life, when scoring the QOLIBRI scale, means are converted to a 0 to 100 rating scale by subtracting 1 from the mean score and then multiplying it by 25, with a value of 0 indicating the worst possible HRQoL and a value of 100 the best possible HRQoL.

For the general population sample, three items of the original QOLIBRI had to be reworded to remove the reference to a TBI. The fifth item from the scale “Self”, “How satisfied are you with what you have achieved since your brain injury?”, was changed to “How satisfied are you with what you have achieved recently?”. The second item from the scale “Physical”, “How bothered are you by effects of any other injuries you sustained at the same time as your brain injury?”, was changed to “How bothered are you by the effects of any injuries you sustained?”. The last item, also assigned to the scale “Physical”, “Overall, how bothered are you by the effects of your brain injury?”, was changed to “Overall, how bothered are you by the effects of any health problems?”.

#### 2.3.2. Sociodemographic and Health Status Data

The sociodemographic and health status data for both samples contained information on sex, age and the highest level of education achieved. In addition, the presence of chronic health conditions (CHC) was recorded for the general population sample, where multiple answers were possible. The question was: “Do you have any of the following chronic health complaints?” Subjects were asked to tick a box for the response options (multiple answers were possible) listed in Table A1.

Additionally, the Glasgow Coma Scale (GCS) was used in the TBI sample to rate TBI severity [36]. A score of 13 to 15 points indicates mild TBI, 9 to 12 moderate TBI, and 3 to 8 severe TBI. The Glasgow Outcome Scale Extended (GOSE), ranging from 1 (death) to 8 (upper good recovery), was used as a measure of recovery status after TBI [37].

### 2.4. Participants

#### 2.4.1. General Population Sample

Participants from the general population sample were recruited by a market research agency (Dynata, Shelton, CT, USA) between 29 June and 31 July 2017. To obtain a representative sample, participants were invited until the required quotas for age, sex and level of education had been achieved. Due to the self-reported nature of the data collection, the sex of participants was collected as gender (male, female). Since gender/sex corresponds to the biological categories of males and females, the word “sex” will be used for consistency and to avoid any confusion. Comparison of the quotas with demographic information obtained from the Organization for Economic Cooperation and Development databank (OECD) [38] and Eurostat database [39] revealed a widely comparable distribution of the groups. Within this online survey based on self-report, the data were collected in Italy, the Netherlands, and the UK. The reference values of the QOLIBRI for the Netherlands and the UK have already been published [40].

**Figure 1:**
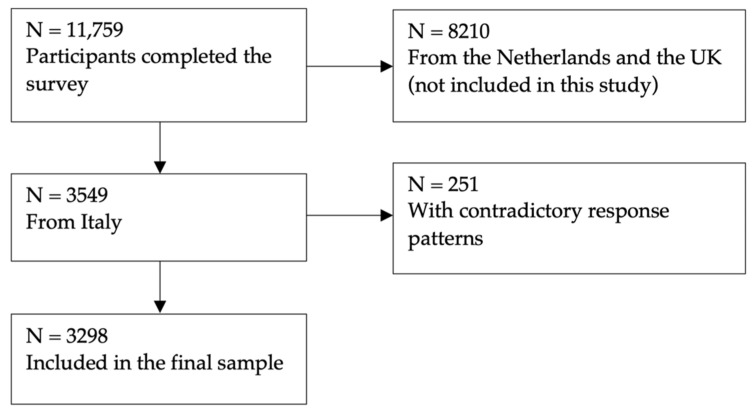
General population sample attrition chart.

In order to increase the representativeness of the sample, Dynata deployed a variety of methods to engage people with diverse motivations to take part in research and to reach participants with different socioeconomic statuses. To avoid self-selection bias, specific details of the project were not visible at the time of the invitation. The project details were only disclosed later on. Participants who answered the survey in less than five minutes were automatically excluded from the analysis. Additionally, participants with contradictory response patterns were excluded. For the QOLIBRI, the following answers were excluded as they were contradictory: If someone chose responses at either the left or right extremes of the Likert scale, that meant that they were not satisfied at all, but also not bothered at all. All collected data were anonymized. The nonresponse rate of the survey was 14.1%. Figure 1 shows the general Italian population sample attrition.

#### 2.4.2. TBI Sample

**Figure 2:**
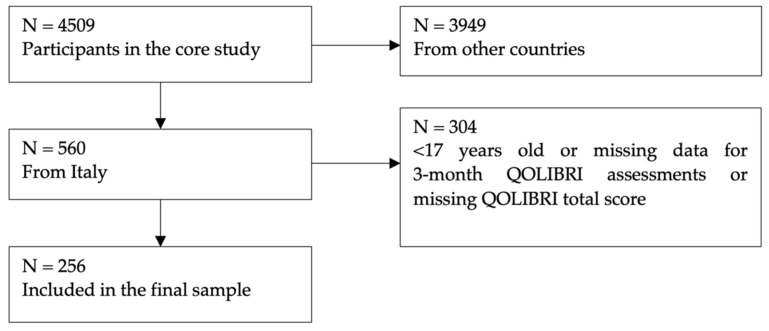
TBI sample attrition chart.

Participants in the TBI sample were a part of the CENTER-TBI study (EC grant 602150), which collected data from 4509 patients in 18 countries [35]. The following inclusion criteria had to be fulfilled: a clinical diagnosis of TBI, presentation in the hospital fewer than 24 h after injury, and an indication for computed tomography (CT). Data were collected between 9 December 2014 and 17 December 2017 via face-to-face visits, in hospital visits, via telephone interviews, or a combination of telephone interview and e-mail. Data on sex, age, time since injury and education was collected at study enrollment based on medical records and self-report. The information on age at study enrollment reflects the age at injury. The QOLIBRI data used was obtained around three months post-injury (i.e., minus two to plus five weeks). Figure 2 shows the TBI sample attrition. No participants with contradictory response patterns were identified. Therefore, all were included in the analyses.

### 2.5. Statistical Analyses

The following section describes the statistical analyses in detail. All the analyses were carried out using R version 4.0.3 [41] employing the packages lavaan [42] and semTools [43] for the calculation of Confirmatory Factor Analysis (CFA) and MI, respectively. The significance level was set at 5%.

#### 2.5.1. Item and Scale Characteristics of QOLIBRI in General Population

Firstly, the item characteristics of the reworded QOLIBRI were examined. This included means, standard deviations, skewness, and a check of the floor and ceiling effects. Skewness was characterized as symmetric for values from −0.5 to 0.5, moderately skewed from ±0.5 to ±1, and highly skewed for values above ±1 [44]. On the scale level, the internal consistency of items was calculated using Cronbach’s alpha. Then, the correlation between scales and the range of correlations between items and their home scales were checked. In order to evaluate the ceiling effects, a cut-off value of 40% was chosen for the highest category “very”. This is twice as high as the 20% that could be expected by chance with five categories. For the floor effects, we controlled by combining the response categories “not at all” and “slightly”, with a cut-off of 10%. The recommendations of the World Health Organization Quality of Life (WHOQOL) Group [45] were followed to exclude items with a Corrected Item-Total Correlation (CITC) higher than 0.4. However, no items had to be excluded.

#### 2.5.2. Construct Validity of QOLIBRI in General Population

We used confirmatory factor analysis (CFA) to verify whether the six-factor structure of the original questionnaire could be replicated for the adapted QOLIBRI applied in the general reference population sample. For this purpose, we first estimated three models: a one-factor model, a two-factor model, and the original six-factor model. The one-factor model assumed a general factor HRQoL that is associated equally with all 37 QOLIBRI items. The two-factor model assumed two intercorrelated factors, where one factor included items from the QOLIBRI that represented satisfaction with certain aspects of an individual’s life (Part A) and the second factor reflected feeling bothered with some aspects of one’s life (Part B). The six-factor model which was described above in detail comprised six factors (Cognition, Self, Daily Life and Autonomy, Social Relationships, Emotions and Physical Problems). Finally, the models were compared using chi-square difference tests.

#### 2.5.3. Measurement Invariance between Samples

The examination of the MI included analyses of individual responses from both samples. Due to the limited sample size in the TBI sample, we had to dichotomize the response categories of the QOLIBRI, with the response categories “not at all” and “slightly” forming the lower category and the response categories “moderately”, “quite” and “very” the higher category. We therefore followed the approach of Wu and Estabrook [46] when testing MI for dichotomized response categories. We estimated increasingly constrained models and compared the model fit among these. We first estimated the baseline model, which is mostly equivalent to configural MI and freely estimates all four parameters (thresholds, loadings, intercepts and residuals). Here, the requirement of configural MI is satisfied when the same number of factors and the same pattern of loadings are equal for both groups. We then estimated the second model, where three parameters are restricted and the thresholds are freely estimated, which corresponds to partial MI. Finally, in the last model, all four parameters were restricted, which is equivalent to full MI.

#### 2.5.4. Regression Analysis

Research suggests that age [47], gender [48], education [49] and the presence of chronic health conditions (CHC) [50,51] have an impact on HRQoL. Therefore, to generate reference values that represent HRQoL for meaningful subgroups, we investigated the influence of these factors on HRQoL as measured by the QOLIBRI total score using multiple linear regression. Available information from the general population sample on these variables and their interactions was included in the regression model. Age was binned into six ordered age categories (18 to 24; 25 to 34; 35 to 44; 45 to 54; 55 to 64; older than 65 years). Bearing in mind that the age—in the form of 10-year age bins—had a significant influence on the total score of the short form of the QOLIBRI, its overall scale—QOLIBRI-OS, in the Italian population [52], the same age bins were used here. Sex was categorized as female and male. Education was assessed as the highest level of education and categorized as one of the following three: low (primary school), middle (diploma, secondary school, high school, or post-high school), or high (college or university). Participants were categorized in terms of CHCs either being present (when they reported at least one CHC) or being absent. The dependent variable was the participants’ QOLIBRI total score.

#### 2.5.5. Reference Values from the General Population Sample

Based on the results of a linear regression analysis, tables were presented with population reference values in form of percentiles (2.5%, 5%, 16%, 30%, 40%, 50%, 60%, 70%, 85%, 95% and 97.25%). Values below the 16th percentile and above the 85th percentile (both rounded up to the next integer) represent low and excellent HRQoL, respectively. These can be used to evaluate whether an individual’s QOLIBRI total score is below, equal to, or above the value of the respective reference group.

## 3. Results

The sociodemographic characteristics of the general population sample are presented in Table 1. Both sexes were represented equally. The mean age of this sample was 45.27 (SD = 14.85) years. Slightly more than a half of the participants (53.97%) reported no CHCs. Detailed information on specific CHCs per age group can be found in Appendix A, Table A1.

Sociodemographic and clinical characteristics of the TBI sample are presented in Table 2. The mean age was 50.63 (SD = 20.75) years and 68.8% of the TBI sample were males. Most subjects (55.94%) had an intermediate level of education. The majority of the TBI sample sustained a mild TBI. Over half of the participants (53.73%) recovered well after TBI.

### 3.1. Item and Scale Characteristics of QOLIBRI in the General Population

Item characteristics including mean value, skewness, and floor and ceiling effects are presented in Table 3. On average, individuals were rather satisfied with their HRQoL (M = 3.62 [3.11–4.02]). The lowest satisfaction scores related to questions on anger or aggression (M = 3.11, SD = 1.26) and the highest satisfaction scores were reported in connection with the ability to find one’s way around (M = 4.02, SD = 0.98), the ability to get out and about (M = 4.02, SD = 1.02), and the ability to carry out domestic activities (M = 4.02, SD = 0.97). With skewness values from 0 to ±0.99, the item distribution can be considered as moderately skewed. None of the satisfaction items from the Part A exceeded the cut-off value for ceiling effects. The reversed scales “Emotions” and “Physical Problems” in Part B, containing bothered items, showed higher values, indicating that individuals from the general population sample were mostly not bothered by problems present in the TBI population. The scales “Cognition” and “Physical Problems” were below the cut-off value of 10%, indicating that the healthy population sample had very few problems in these domains.

Table 4 provides Cronbach’s alpha characterizing the internal consistency of the six QOLILBRI scales. Coefficients ranged from 0.87 to 0.92 indicating good to excellent internal consistency of the QOLIBRI scales [53]. Based on corrected item-total correlations (CITC) and the cut-off of 0.40, all items were considered consistent. The subscales were moderately to highly intercorrelated (*r* between 0.35 and 0.77). The highest correlation was found between the subscales “Daily Life and Autonomy” and “Self” (r = 0.83), while the lowest correlation was between the scales “Emotions” and “Cognition” (r = 0.35).

### 3.2. Construct Validity of the QOLIBRI in the General Population

In order to evaluate the latent factor structure of the adapted QOLIBRI, CFAs were carried out, comparing the one, two and six factorial models. Table 5 summarizes the goodness of fit indices for these models, showing the best fit for the six factorial model with χ2(614) = 7473, *p* < 0.001, CFI = 0.994, and RMSEA = 0.058, 90% CI (0.057; 0.059) [54].

### 3.3. Measurement Invariance

The results of the MI analyses indicated no significant difference between the configural and partial invariance models (Table 6), thus partial invariance can be assumed. However, a comparison of the partial and full invariance models revealed statistically significant differences, indicating that thresholds differed between these models. Further analysis has been undertaken to assess the practical significance of these differences. Examining the thresholds in the partial invariance model showed that these values differed between the general population sample and the TBI sample (Table A2), indicating that the response behavior was not identical in both groups. However, these threshold differences did not exceed 5%. Therefore, the difference between partial and full measurement invariance can be interpreted as being non-significant, resulting in full measurement invariance between the TBI and general population sample. Thus, when comparing QOLIBRI scores between general population and TBI population samples, the differences in scores can be attributed to real differences in HRQoL.

### 3.4. Linear Regression Analysis

Regression analysis revealed a significant impact of age, CHCs and education (Table 7). Individuals in all other age groups displayed significantly higher QOLIBRI scores than individuals aged 18 to 24 years. The presence of a CHC significantly influenced HRQoL, since healthy individuals had higher QOLIBRI scores than individuals with at least one chronic health condition. Individuals with a high, but not those with a medium level of education had significantly higher QOLIBRI scores than individuals with lower education. The effect of sex or any other interaction did not significantly contribute to explaining the QOLIBRI scores.

### 3.5. QOLIBRI Reference Values for the Italian General Population

Based on the results of the regression analysis, reference values were stratified by age, level of education, and the presence of at least one CHC (Table 8). Additionally, we stratified reference values by sex because prior research on HRQoL in individuals after TBI indicates sex effects on HRQoL [55,56,57]. Reference values without categorization by sex can be found in the Appendix A (s. Table A3). Reference tables for the QOLIBRI subscales can be found in Appendix A (s. Table A4, Table A5, Table A6, Table A7, Table A8 and Table A9).

The following example will try to illustrate how to use these values. After a TBI, a 50-year-old woman with diabetes presented with a QOLIBRI total score of 65. The appropriate reference values are those of females with at least one CHC in the age group of 45 to 54 years (Table 8). Table 8 shows that about 65% of individuals in her age group reported the same or a lower level of HRQoL. Her value lies in the range of one standard deviation above the median and can thus be considered as being average. Based on the 16%-percentile cut-off value, HRQoL is interpreted as being below average for female individuals of 50 years with one CHC when the QOLIBRI total score is lower than 42.

## 4. Discussion

The aim of this study was to provide reference values for the QOLIBRI derived from a general Italian population sample. For that purpose, some conditions had to be fulfilled. First, CFA was used to verify that the adjusted QOLIBRI had the assumed six-factorial structure (Cognition, Self, Daily Life and Autonomy, Social Relationships, Emotions, Physical Problems) like the original QOLIBRI version for adults after TBI. This requirement was met and the results were almost consistent with an earlier study [40] that applied the adapted QOLIBRI questionnaire to general population samples from the Netherlands and the UK.

Gorbunova et al. [40] showed that in the Dutch population, the interaction between gender and CHCs was also significant in the regression analysis. This was not the case in the Italian or in the United Kingdom populations. Concerning the QOLIBRI total score without further stratification, a value below 50 obtained from general Italian sample indicates impaired HRQoL. The values obtained from the English and Dutch general population samples were lower (i.e., 44 for the UK) and higher (i.e., 55 for the Netherlands), respectively [40]. Since no differences can be observed in terms of the distribution of the sociodemographic or health-related factors, these findings can be explained by the differences in HRQoL across the countries [58,59,60,61]. For example, Alonso et al. [61] found that participants from the Netherlands (M = 55.2) reported the highest generic mental HRQoL score as measured using the Short Form-36 (SF-36) mental component summary score, compared with other countries (e.g., Italy: M = 50.3). In addition, the European Study of Epidemiology of Mental Disorders within six countries found that the proportion of respondents reporting problems on any of the EuroQol-5 Dimensions (EQ-5D) [62] was significantly higher in France and lower in Spain and Italy [58]. Taken together, all these differences emphasize the importance of country-specific reference values, which is also the case for TBI-specific HRQoL assessments.

The MI analyses indicated that the same construct was measured in the general Italian reference sample and in the TBI population. Although the full MI model differed from the partial MI model in terms of model fit, analyses of threshold fluctuations indicated that thresholds did not differ more than 5% and were thus negligible. The same conclusion could also be drawn for the QOLIBRI in the Dutch and UK samples [40,58,59,60,61].

Our results showed that younger age, presence of CHCs, and lower level of education are associated with worse HRQoL measured using the QOLIBRI. Wu et al. [52] found similar results for the use of the short version of the QOLIBRI, the QOLIBRI-OS, in an Italian general population sample providing reference values. The use of the same age bins in calculating the regression analyses presented, as well as in stratifying the reference values, ensures that the reference values of the two instruments are comparable in the future. Regarding age differences, a study examining HRQoL after heart failure found that older patients’ HRQoL exceeded expectations for their age, whereas younger individuals complained of loss of activities or roles and rated their HRQoL as being correspondingly worse. The authors suggested that better HRQoL in older compared with younger patients was due to the older patients’ ability to reconceptualize their expectations in relation to their health problems. Duke et al. [63] also demonstrated that older people who had adapted their activities to the chronic illness in question had better mental health, suggesting that it is not just the presence of health problems or young age that determines good quality of life.

In addition, it should be noted that sex did not play a role either in the study by Wu et al. [52] nor in the present study. However, the literature on TBI regarding sex or gender differences is inconsistent [57,64,65,66], while there is strong evidence that gender represents an influential factor in TBI [67]. Previous research shows that sex differences were found to possibly affect sustaining a TBI [68], to impact post-concussion symptoms [56,69], depression [70], anxiety [70], as well as recovery after TBI [71,72]. A recent study by Mikolic et al. (2021), examining differences between men and women in treatment and outcome after TBI, finds that after mild TBI women reported lower generic and disease-specific HRQoL than men. Despite controversial research findings, gender/sex seems to be important for outcome assessment after TBI. Therefore, we have also added a stratification of reference values by sex in addition to the stratification by age, presence of CHC and education.

In contrast to our TBI sample showing a negative association between the HRQoL and age (*r* = −0.18), as well as to prior research that has found a decrease in HRQoL in older subjects with a TBI history [47], the general population sample investigated in the present study displayed higher HRQoL with increasing age. This is in line with findings from a non-TBI Taiwanese sample, which showed a positive effect of age on mental HRQoL and negative influence on physical HRQoL measured using the generic Short Form 12 (SF-12) [73]. In our sample, we used the QOLIBRI total score, which incorporates both mental and physical aspects of HRQoL. Further research should investigate the differential effects of age on individual QOLIBRI dimensions.

It is reasonable to assume that chronic health problems have an influence on HRQoL [74]. Our results showed that individuals with CHCs exhibited lower QOLIBRI total scores than individuals without CHCs. These results are consistent with previous research which indicates an inverse relationship between CHCs and HRQoL [75].

In addition, level of education was also associated with better HRQoL. Individuals with a higher education level reported higher QOLIBRI total scores in comparison to individuals with low education levels. These findings are in line with prior research showing an association between higher education levels and better HRQoL in non-TBI [76] and TBI [77,78] populations. The relationship between education and HRQoL can likely be explained by the opportunities higher education and better socioeconomic status provide, furthering, for example, self-determination through better income and better access to health services [79,80,81].

### 4.1. Strengths and Limitations

The most important strength of our study is the number of survey participants, which allowed reference values to be calculated stratified by several sub-groups. For example, we were able to provide reference tables for the individuals with and without CHCs and integrating the education levels. The interpretation of HRQoL for Italian individuals after TBI has thereby been improved. Furthermore, reference values based on percentiles are a common approach in clinical practice, facilitating the interpretation and communication of the QOLIBRI scores. The comparison with a (healthy) general population improves the comprehensibility of the test results for the patients.

This study also has several limitations that may require discussion. The first limitation concerns the recruitment of the general population sample. Recruitment was carried out via online platforms and strived for maximum representativeness. However, the online nature of the recruitment only captures certain population groups, such as only those who have Internet access, which may have led to selection biases [82]. In addition, we do not have information about those who declined the survey invitation, which is one of the main issue of online surveys [83]. Possible carelessness in answering online surveys [84] as well as the lack of opportunity to verify the authenticity of the data are notable limitations [82]. Moreover, the severity of the CHCs, as well as their duration, were not recorded because the analyses of these characteristics were beyond the scope of this study. Future studies may investigate the influence of these factors on disease-specific HRQoL.

With respect to the TBI sample, it should be noted that its relatively small size made a dichotomization of the QOLIBRI’s response categories necessary, which always results in a loss of information [85,86]. Furthermore, the vast majority of the TBI sample (71%) consisted of mild TBI, which could have led to response categories not being exhausted (e.g., not at all satisfied or very bothered), requiring the modification of the number of response categories for MI analysis. However, this limitation only concerns the comparison of the QOLIBRI between the general and the TBI sample. To fill this gap, future research should investigate potential differences between Italian TBI and general population samples employing larger TBI samples. With regard to injury severity in the TBI sample, it should be noted that 13.7% of subjects had missing information, which is common in clinical trials. These missing data were not imputed since this information has not been used in the further analyses. The 13.7% of missing values for education were either due to the fact that the level of education was unknown or not reported. Since we did not include any of the above variables in determining the reference values and used them only for the descriptive statistics of the TBI sample, the missing values had no further impact on our results.

The QOLIBRI is an internationally widely used instrument, which has been translated into 26 languages. The reference values for the Italian population presented here may help to consider cultural differences in HRQoL. In addition to the total score, reference values on the subscale level allow the HRQoL domains to be evaluated more precisely. However, to date, there are reference values only for two further countries (i.e., the Netherlands and the UK). Therefore, further studies are required that investigate country-specific reference values for the QOLIBRI in the general population to enable multinational studies on TBI supporting the understanding of the clinical meaning of HRQoL after TBI.

### 4.2. Conclusions

This study contributes to TBI outcome research by providing reference values for the TBI-specific instrument QOLIBRI for an Italian general population stratified by age, education, gender, and the presence of CHCs. Researchers and clinicians are now able to employ reference values for individuals from Italy which could help them to better interpret HRQoL after TBI in individuals and to adjust their treatment accordingly, which in turn could help to improve the quality of life of the individuals concerned.

## Figures and Tables

**Table 1:** Characteristics of the general population sample (N = 3298).

Age (years)	Mean	SD	Range
	45.27	14.85	57
	Group	N	%
Sex	Male	1649	50
	Female	1649	50
Education level	Low	1111	33.69
	Middle	1845	55.94
	High	342	10.37
Number of chronic health complaints	None	1780	53.98
	One	948	28.74
	Two and more	570	17.28

Note: N = number of cases, % = relative frequencies, SD = Standard deviation, low = primary school; middle = diploma, secondary school, high school, or post-high school; high = college or university.

**Table 2:** Characteristics of the TBI sample (N = 256).

Age (years)	Mean	SD	Range
	50.63	20.75	75
	Group	N	%
Sex	Male	176	68.8
	Female	80	31.2
Education level	Low	4	1.6
	Middle	166	64.8
	High	51	19.9
	Missing	35	13.7
	Mild	182	71.1
TBI severity (GCS)	Moderate	27	10.5
	Severe	47	18.4
	Missing	35	13.7
Recovery status (GOSE) at 3 months post injury	Good recovery (7–8)	135	53.7
	Moderate disability (5–6)	52	20.3
	Severe disability (2/3–4)	69	27.0

Note: N = number of cases, % = relative frequencies, SD = Standard deviation, GCS = Glasgow Coma Scale, GOSE = Glasgow Outcome Scale Extended.

**Table 3:** Item characteristics of the QOLIBRI in the general population.

	Mean	SD	Skewness	% in Cat. “Very”	% in Cat. “Not at All” and “Slightly”
Cognition					
Concentrate	3.83	0.93	−0.79	23.4	8.2
Expressing yourself	3.97	0.91	−0.87	29.6	6.9
Memory	3.78	0.92	−0.63	21.3	8.7
Plan and problem solving	3.95	0.91	−0.87	28.6	7.2
Decisions	3.96	0.94	−0.87	30.9	7.1
Navigate	4.02	0.98	−0.99	36.3	7.7
Speed of thinking	4.00	0.87	−0.83	30.4	5.4
Self					
Energy	3.59	0.95	−0.63	14.5	12.1
Motivation	3.66	0.99	−0.67	18.8	12.0
Self-esteem	3.53	1.08	−0.60	18.1	16.6
Appearance	3.38	1.07	−0.54	12.1	19.1
Achievements	3.46	1.05	−0.57	14.3	16.6
Self-perception	3.52	1.04	−0.63	15.2	15.9
Future	3.17	1.14	−0.39	10.0	25.0
Daily Life and Autonomy					
Independence	3.79	1.10	−0.76	30.1	12.5
Get out and about	4.02	1.02	−0.98	38.7	9.0
Domestic activities	4.02	0.97	−0.92	36.2	7.4
Run personal finances	3.77	1.04	−0.78	26.0	11.0
Participation at work	3.76	1.00	−0.74	23.6	10.7
Social and leisure activities	3.47	1.09	−0.51	17.1	18.5
In charge of life	3.67	1.04	−0.69	21.6	12.9
Social Relationships					
Affection towards others	3.92	0.99	−0.87	31.0	8.0
Family	3.86	1.01	−0.89	28.4	9.8
Friends	3.69	1.03	−0.75	21.3	12.8
Partner	3.71	1.20	−0.82	29.9	15.6
Sex life	3.39	1.27	−0.54	20.1	23.0
Attitudes of others	3.45	1.04	−0.58	13.1	16.9
Emotions					
Loneliness	3.48	1.24	−0.24	6.1	48.2
Boredom	3.22	1.25	−0.06	8.7	42.1
Anxiety	3.14	1.34	−0.03	13.1	40.7
Sadness	3.14	1.38	−0.04	14.3	41.3
Anger/Aggression	3.11	1.26	0.00	11.2	37.8
Physical Problems					
Slow/clumsiness	3.80	1.24	−0.62	4.9	60.5
Effects other injuries	3.72	1.15	−0.51	3.7	59.2
Pain	3.21	1.22	−0.14	9.2	42.7
Seeing/hearing	3.54	1.24	−0.37	6.1	53.4
Effects health problems	3.30	1.20	−0.21	8.2	44.7

**Table 4:** Psychometric properties of the QOLIBRI scales in general population.

	Cronbach’s Alpha	Item-Total Correlation Range	Correlations between Subscales Scores				
QOLIBRI Domains			(1)	(2)	(3)	(4)	(5)
(1) Cognition	0.91	0.67–0.81	1				
(2) Self	0.92	0.69–0.89	0.69	1			
(3) Daily Life and Autonomy	0.90	0.68–0.80	0.77	0.83	1		
(4) Social Relationships	0.88	0.71–0.79	0.64	0.76	0.76	1	
(5) Emotions	0.87	0.62–0.87	0.35	0.42	0.38	0.39	1
(6) Physical Problems	0.88	0.66–0.83	0.38	0.43	0.42	0.31	0.55

**Table 5:** Results of confirmatory factor analyses of the QOLIBRI in general population.

Model Comparison							
Model	CFI	RMSEA (90% CI)	χ2 (df)	*p*	Comparison between Models	∆χ2 (∆df)	*p*
One-factor	0.932	0.187 (0.186; 0.188)	73,414 (629)	<0.001			
Two-factor	0.972	0.120 (0.119; 0.122)	30,633 (628)	<0.001	One- vs. Two-factor	3009.9 (1)	<0.001
Six-factor	0.994	0.058 (0.057; 0.059)	7473 (614)	<0.001	Two- vs. Six-factor	3496.9 (14)	<0.001

Note: CFI: scaled Comparative Fit Index (Cut-off: CFI > 0.95); RMSEA (90% CI, Value for adequate/regular model fit: 0.05 < RMSEA < 0.08): scaled root mean square error of approximation with 90% confidence interval; χ2: scaled chi-square statistics; df: scaled degrees of freedom; *p*: *p*-value of chi-square (difference) statistics; ∆χ2: difference in chi-square statistics under Sattora–Bentler (2001) correction; ∆df: difference in degrees of freedom.

**Table 6:** Results of Measurement Invariance testing: Model comparison.

Model Comparison							
Model	CFI	RMSEA (90% CI)	χ2 (df)	*p*	Comparison between (Invariance Models)	∆χ2 (∆df)	*p*
Configural	0.986	0.030 (0.028; 0.031)	3151.63 (1228)	<0.001			
Partial	0.988	0.026 (0.025; 0.028)	2795.62 (1253)	<0.001	configural vs. partial	7.94 (25)	0.999
Full	0.988	0.027 (0.025; 0.028)	2918.75 (1290)	<0.001	partial vs. full	92.95 (37)	<0.001

Note: CFI: scaled Comparative Fit Index (Cut-off: CFI > 0.95); RMSEA (90% CI, Value for adequate/regular model fit: 0.05 < RMSEA < 0.08): scaled root mean square error of approximation with 90% confidence interval; χ2: scaled chi-square statistics; df: scaled degrees of freedom; *p*: *p*-value of chi-square (difference) statistics; ∆χ2: difference in chi-square statistics under Sattora–Bentler (2001) Correction; ∆df: difference in degrees of freedom; Identification constraints for the invariance models: Configural: item intercepts = 0, residual variances = 1, latent factor means = 0, latent factor variances = 1; Partial: item intercepts = 0, residual variances = 1. Only in the reference group latent factor means = 0 and variances = 1; Full: item intercepts = 0, residual variances = 1. Only in the reference group factor means = 0, factor variances = 1.

**Table 7:** Results of the linear regression analysis.

Predictors and Interactions	Reference Group	β	SE
Intercept		63.30 *	1.21
Age (25–34)	Age (18–24)	1.58	1.38
Age (35–44)		4.64 *	1.32
Age (45–54)		7.16 *	1.39
Age (55–64)		9.22 *	1.45
Age (≥65)		12.53 *	1.58
Sex (female)	Sex (male)	−1.04	0.74
CHC (yes)	CHC (no)	−7.66 *	1.91
Education (middle)	Education (low)	1.16	0.59
Education (high)		1.98 *	0.97
Sex (female) × CHCs (yes)	Sex (male) × CHCs (yes)	−0.56	1.08
Age (25–34) × CHCs (yes)	Age (18–24) × CHCs (yes)	−3.51	2.27
Age (35–44) × CHCs (yes)		−2.12	2.17
Age (45–54) × CHCs (yes)		−3.44	2.19
Age (55–64) × CHCs (yes)		−1.91	2.24
Age (≥65) × CHCs (yes)		−1.37	2.37

Note: β indicates an unstandardized regression coefficient (slope); SE, standard error; CHC, Chronic Health Condition; * Significant at *p* < 0.05.

**Table 8:** Reference values for the QOLIBRI total score obtained from the general population sample in Italy stratified by sex, health status, age, and education.

Sex × Health Status × Age				Low HRQoL		−1 SD			Md			+1 SD	High HRQoL	
Sex	Health Status	Age	N	2.5%	5%	16%	30%	40%	50%	60%	70%	85%	95%	97.25%
Female	Healthy	18–24	82	38	43	50	57	61	63	66	69	78	94	96
		25–34 35–44 45–54 55–64	159 201 167 136	32 39 42 43	40 43 47 46	51 50 54 55	57 59 64 64	62 64 68 68	66 68 71 73	70 74 76 76	74 79 79 79	82 84 88 86	92 93 98 93	99 95 100 95
		≥65	89	49	52	58	68	73	76	82	88	93	99	100
	At least one CHC	18–24	63	22	27	41	46	48	51	55	58	70	80	86
		25–34 35–44 45–54 55–64	125 161 173 169	18 25 28 28	25 31 32 31	37 42 42 47	49 50 51 56	51 54 55 59	54 57 59 61	58 62 63 65	62 66 69 71	73 76 78 78	85 82 87 87	88 85 90 93
		≥65	124	38	41	50	58	62	68	72	77	83	91	97
Male	Healthy	18–24	117	44	46	50	55	60	65	67	71	79	88	94
		25–34 35–44 45–54 55–64	187 242 170 134	40 41 44 50	44 45 48 53	51 52 56 63	58 61 62 68	61 67 67 72	65 71 71 75	68 74 75 79	72 79 80 83	82 84 87 91	89 95 96 98	96 98 99 100
		≥65	96	56	58	65	72	74	77	79	83	89	99	100
	At least one CHC	18–24	48	30	37	47	51	54	60	63	67	77	87	93
		25–34 35–44 45–54 55–64	94 137 154 147	22 28 25 29	33 36 29 41	42 45 45 49	48 52 51 58	50 56 55 62	52 60 59 66	55 64 65 71	60 68 69 75	69 75 78 80	81 84 88 86	87 90 90 89
		≥65	123	41	43	56	61	66	69	73	77	83	91	92
Sex × Health Status × Education				Low HRQoL		−1 SD			Md			+1 SD	High HRQoL	
Sex	Health Status	Education	N	2.5%	5%	16%	30%	40%	50%	60%	70%	85%	95%	97.25%
Female	Healthy	Low	321	39	43	50	58	63	67	72	77	84	94	100
		Middle	445	39	45	53	61	65	71	75	79	88	95	99
		High	68	43	51	59	65	70	74	79	82	87	92	95
	At least one CHC	Low	296	28	32	42	49	54	58	63	68	78	86	89
		Middle	439	24	28	43	52	55	59	63	69	79	87	94
		High	80	38	39	48	55	57	62	65	72	78	84	85
Male	Healthy	Low	270	39	45	51	59	65	69	73	79	86	93	96
		Middle	576	44	49	55	63	67	71	75	79	86	96	100
		High	100	43	48	55	61	65	68	74	78	85	97	99
	At least one CHC	Low	224	33	39	49	55	60	63	67	71	78	87	90
		Middle	385	27	34	46	52	57	61	67	70	79	87	92
		High	94	16	36	44	50	53	57	63	67	79	85	92
		Total	3298	32	38	50	56	61	66	70	75	83	92	97

Note: HRQoL: Health-Related Quality of Life; 50% percentiles represent 50% of the distribution corresponding to the median (*Md*); *SD*: Standard Deviation; values from −1 standard deviation (16%) to +1 standard deviation (85%) are within the regular range (i.e., not impaired HRQoL). Values below 16% denote low HRQoL and values above 85% indicate outstanding HRQoL.

## Data Availability

All relevant data are available upon request from CENTER-TBI, and the authors are not legally allowed to share it publicly. The authors confirm that they received no special access privileges to the data. CENTER-TBI is committed to data sharing and in particular to responsible further use of the data. Hereto, we have a data sharing statement in place: https://www.center-tbi.eu/data/sharing (accessed on 1 July 2022). The CENTER-TBI Management Committee, in collaboration with the General Assembly, established the Data Sharing policy, and Publication and Authorship Guidelines to assure correct and appropriate use of the data as the dataset is hugely complex and requires help of experts from the Data Curation Team or Bio- Statistical Team for correct use. This means that we encourage researchers to contact the CENTER-TBI team for any research plans and the Data Curation Team for any help in appropriate use of the data, including sharing of scripts. Requests for data access can be submitted online: https://www.center-tbi.eu/data (accessed on 1 July 2022). The complete Manual for data access is also available online: https://www.center-tbi.eu/files/SOP-Manual-DAPR-2402020.pdf (accessed on 1 July 2022).

## Supplementary Materials

The following supporting information can be downloaded at: https://www.mdpi.com/article/10.3390/jcm12020491/s1, Membership of CENTER-TBI Participants and Investigators is provided in the Supplementary Materials.

## Appendix A

**Table A1:** Prevalence of CHC per age group.

CHC (N)	18–24 (n = 310)	25–34 (n = 565)	35–44 (n = 741)	45–54 (n = 664)	55–64 (n = 586)	<65 (n = 432)	Total (n = 1518)
Asthma	26	48	60	41	41	26	242
Heart Disease	2	7	5	10	18	20	62
Stroke	5	6	5	10	5	7	38
Diabetes	13	25	34	50	52	57	231
Back Complaints	12	31	43	50	43	28	207
Arthritis	6	32	49	64	101	68	320
Rheumatism	4	30	43	51	55	37	220
Cancer	5	6	11	11	11	18	62
Memory Problems due to Dementia	4	13	13	11	11	2	54
Memory Problems due to Ageing	5	7	11	25	47	44	139
Depression	57	95	111	105	81	50	499
Other	19	35	65	89	80	56	344

**Table A2:** Response probabilities estimated for general population sample from the full invariance model in comparison to TBI sample.

	General Population (TBI as a Ref.)
COGNITION	Thresholds
Concentrate	0.704 (0.000)
Expressing yourself	0.757 (0.000)
Memory	0.670 (0.004)
Plan and problem solving	0.753 (0.000)
Decisions	0.742 (−0.001)
Navigate	0.754 (−0.004)
Speed of thinking	0.766 (0.002)
SELF	
Energy	0.597 (0.009)
Motivation	0.628 (0.004)
Self-esteem	0.576 (0.003)
Appearance	0.518 (0.000)
Achievements	0.541 (−0.011)
Self-perception	0.580 (−0.001)
Future	0.435(−0.004)
DAILY LIFE AND AUTONOMY	
Independence	0.656 (−0.001)
Get out and about	0.745 (0.002)
Domestic activities	0.750 (0.002)
Run personal finances	0.660 (−0.005)
Participation at work	0.662 (0.001)
Social and leisure activities	0.546 (0.002)
In charge of life	0.628 (−0.002)
SOCIAL RELATIONSHIPS	
Affection towards others	0.716 (0.000)
Family	0.709 (−0.001)
Friends	0.649 (−0.002)
Partner	0.649 (0.000)
Sex life	0.547(0.007)
Attitudes of others	0.544 (−0.003)
EMOTIONS	
Loneliness	0.482 (−0.004)
Boredom	0.421 (0.000)
Anxiety	0.407 (0.001)
Sadness	0.413 (0.002)
Anger/Aggression	0.378 (0.000)
PHYSICAL PROBLEMS	
Slow/clumsiness	0.605 (0.006)
Effects other injuries	0.592 (0.011)
Pain	0.427 (−0.009)
Seeing/hearing	0.534 (−0.005)
Effects health problems	0.447 (−0.004)

Note: For measurement invariance testing with TBI samples response categories “not at all” and “slightly” were recorded as 1.

**Table A3:** Reference values for the QOLIBRI total score obtained from the general population sample in Italy stratified by health status, age, and education.

Health Status × Age													
			Low HRQoL		−1 SD			Md			+1 SD	High HRQoL	
Health Status	Age	N	2.5%	5%	16%	30%	40%	50%	60%	70%	85%	95%	97.25%
Healthy	18–24	199	39	45	50	56	61	64	67	70	79	91	95
	25–34	346	37	42	51	57	62	65	69	73	82	91	98
	35–44	443	40	44	51	60	65	70	74	79	84	94	98
	45–54	337	43	48	55	62	67	71	76	80	88	97	100
	55–64	270	44	48	59	66	72	74	77	82	88	96	100
	≥65	185	52	55	62	70	74	77	81	85	92	99	100
At least one CHC	18–24	111	24	32	44	48	51	53	58	64	72	85	91
	25–34	219	18	27	38	48	50	53	56	61	72	84	89
	35–44	298	25	35	43	51	55	58	63	67	75	83	87
	45–54	327	27	31	42	51	55	59	64	69	78	87	90
	55–64	316	28	32	49	56	60	64	68	73	79	87	92
	≥65	247	39	42	52	59	63	69	72	77	83	91	94
Health Status × Education													
			Low HRQoL		−1 SD			Md			+1 SD	High HRQoL	
Health Status	Education	N	2.5%	5%	16%	30%	40%	50%	60%	70%	85%	95%	97.25%
Healthy	Low	591	39	43	50	58	64	68	73	78	85	93	98
	Middle	1021	42	47	55	62	67	71	75	79	87	96	100
	High	168	41	49	55	64	67	71	75	80	86	95	99
At least one CHC	Low	520	30	35	45	52	56	61	65	70	78	87	89
	Middle	824	25	30	44	52	56	60	65	70	79	87	92
	High	174	31	37	46	51	56	60	64	69	79	84	91

Note: HRQoL: Health-Related Quality of Life; 50% percentiles represent 50% of the distribution corresponding to the median (Md); SD: Standard Deviation; values from −1 standard deviation (16%) to +1 standard deviation (85%) are within the regular range (i.e., not impaired HRQoL). Values below 16% denote low HRQoL and values above 85% indicate outstanding HRQoL.

**Table A4:** Reference values for the QOLIBRI Cognition scale obtained from the general population sample in Italy stratified by sex, health status, age, and education.

Sex × Health Status × Age				Low HRQoL		−1 SD			Md			+1 SD	High HRQoL	
Sex	Health Status	Age	N	2.5%	5%	16%	30%	40%	50%	60%	70%	85%	95%	97.25%
Female	Healthy	18–24	82	43	47	50	65	72	75	81	85	93	100	100
		25–34	159	25	32	54	65	72	75	79	83	93	100	100
		35–44	201	36	43	54	68	72	75	79	86	97	100	100
		45–54	167	44	50	65	72	75	75	79	86	97	100	100
		55–64	136	43	50	61	72	75	75	79	86	93	100	100
		≥65	89	44	50	68	75	76	86	90	93	100	100	100
	At least one CHC	18–24	63	29	36	50	58	61	65	68	75	83	93	99
		25–34	125	22	29	43	50	58	68	72	75	86	96	100
		35–44	161	18	25	43	58	65	72	75	79	90	97	100
		45–54	173	34	36	54	65	71	75	75	83	93	100	100
		55–64	169	40	41	61	68	72	75	79	83	93	100	100
		≥65	124	40	50	65	75	75	77	83	86	95	100	100
Male	Healthy	18–24	117	36	50	54	65	72	75	75	83	88	97	100
		25–34	187	36	43	50	68	72	75	75	83	90	100	100
		35–44	242	43	50	56	72	75	75	83	86	97	100	100
		45–54	170	43	50	65	75	75	75	79	86	97	100	100
		55–64	134	50	61	72	75	79	84	90	93	100	100	100
		≥65	96	63	65	72	75	79	83	86	90	100	100	100
	At least one CHC	18–24	48	26	29	50	61	68	75	83	86	90	96	100
		25–34	94	16	24	40	50	50	58	65	72	83	93	97
		35–44	137	25	32	50	61	68	72	75	75	90	97	100
		45–54	154	24	29	49	58	68	72	75	83	93	100	100
		55–64	147	30	44	59	68	73	75	75	83	90	100	100
		≥65	123	40	50	65	72	75	79	83	86	96	100	100
Sex × Health Status × Education				Low HRQoL		−1 SD			MD			+1 SD	High HRQoL	
Sex	Health Status	Education	N	2.5%	5%	16%	30%	40%	50%	60%	70%	85%	95%	97.25%
Female	Healthy	Low	321	40	43	50	65	72	75	75	83	93	100	100
		Middle	445	43	50	61	72	75	75	83	86	97	100	100
		High	68	24	48	71	75	79	83	86	90	97	100	100
	At least one CHC	Low	296	29	36	50	61	68	72	75	83	90	100	100
		Middle	439	25	33	50	65	68	72	75	83	90	100	100
		High	80	50	50	61	72	75	75	79	86	93	100	100
Male	Healthy	Low	270	36	43	54	68	74	75	79	83	93	100	100
		Middle	576	45	50	65	75	75	79	83	86	97	100	100
		High	100	45	50	61	72	75	75	79	86	97	100	100
	At least one CHC	Low	224	28	36	50	65	68	75	75	83	90	100	100
		Middle	385	23	33	50	65	72	75	75	83	90	100	100
		High	94	23	28	47	54	58	68	75	83	90	97	100

Note: HRQoL: Health-Related Quality of Life; 50% percentiles represent 50% of the distribution corresponding to the median (*Md*); *SD*: Standard Deviation; values from −1 standard deviation (16%) to +1 standard deviation (85%) within the regular range (i.e., not impaired HRQoL). Values below 16% denote low HRQoL and values above 85% indicate outstanding HRQoL.

**Table A5:** Reference values for the QOLIBRI Self scale obtained from the general population sample in Italy stratified by sex, health status, age, and education.

Sex × Health Status × Age				Low HRQoL		−1 SD			Md			+1 SD	High HRQoL	
Sex	Health Status	Age	N	2.5%	5%	16%	30%	40%	50%	60%	70%	85%	95%	97.25%
Female	Healthy	18–24	82	22	25	40	50	58	65	71	75	79	100	100
		25–34	159	8	14	36	50	58	65	72	75	87	100	100
		35–44	201	18	25	40	54	61	68	75	75	86	100	100
		45–54	167	25	29	50	64	68	72	75	79	90	100	100
		55–64	136	18	31	50	58	65	72	75	75	85	97	97
		≥65	89	41	45	58	68	72	75	75	83	93	100	100
	At least one CHC	18–24	63	8	11	22	36	43	50	55	61	65	75	79
		25–34	125	8	11	22	40	47	50	58	61	75	82	96
		35–44	161	8	8	29	43	50	54	61	68	75	86	90
		45–54	173	5	11	25	43	50	58	61	68	75	90	96
		55–64	169	11	15	36	50	55	65	68	72	75	90	96
		≥65	124	15	25	40	50	54	61	68	72	79	90	100
Male	Healthy	18–24	117	25	35	50	57	65	68	75	75	86	95	100
		25–34	187	25	29	50	54	61	68	72	75	83	99	100
		35–44	242	22	36	50	58	68	72	75	75	86	100	100
		45–54	170	23	36	50	58	65	72	75	75	86	100	100
		55–64	134	36	46	54	65	72	72	75	79	90	100	100
		≥65	96	36	53	62	68	72	75	75	79	89	100	100
	At least one CHC	18–24	48	11	25	38	50	53	61	68	72	79	94	100
		25–34	94	6	11	25	39	47	50	53	58	75	86	97
		35–44	137	11	22	36	47	54	58	65	72	78	83	90
		45–54	154	11	18	29	47	50	58	65	69	79	90	93
		55–64	147	15	21	43	54	61	65	72	75	79	90	95
		≥65	123	22	26	50	60	65	68	72	75	83	93	93
Sex × Health Status × Education				Low HRQoL		−1 SD			MD			+1 SD	High HRQoL	
Sex	Health Status	Education	N	2.5%	5%	16%	30%	40%	50%	60%	70%	85%	95%	97.25%
Female	Healthy	Low	321	11	25	40	54	61	68	75	75	86	100	100
		Middle	445	22	29	47	58	65	68	75	75	86	100	100
		High	68	28	36	54	65	68	75	75	79	90	100	100
	At least one CHC	Low	296	8	15	29	47	50	54	65	68	75	86	96
		Middle	439	8	11	29	43	50	58	61	68	75	90	97
		High	80	15	22	36	50	56	58	65	68	79	83	86
Male	Healthy	Low	270	25	33	47	54	65	68	75	75	86	100	100
		Middle	576	27	36	50	61	68	72	75	75	86	100	100
		High	100	25	29	50	64	68	72	75	75	86	100	100
	At least one CHC	Low	224	17	25	40	50	58	65	68	72	77	90	98
		Middle	385	11	22	34	47	54	61	65	72	79	89	93
		High	94	6	14	32	43	50	54	65	68	79	93	96

Note: HRQoL: Health-Related Quality of Life; 50% percentiles represent 50% of the distribution corresponding to the median (*Md*); *SD*: Standard Deviation; values from −1 standard deviation (16%) to +1 standard deviation (85%) within the regular range (i.e., not impaired HRQoL). Values below 16% denote low HRQoL and values above 85% indicate outstanding HRQoL.

**Table A6:** Reference values for the QOLIBRI Daily Life and Autonomy scale obtained from the general population sample in Italy stratified by sex, health status, age, and education.

Sex × Health Status × Age				Low HRQoL		−1 SD			MD			+1 SD	High HRQoL	
Sex	Health Status	Age	N	2.5%	5%	16%	30%	40%	50%	60%	70%	85%	95%	97.25%
Female	Healthy	18–24	82	33	43	50	54	65	68	72	75	86	100	100
		25–34	159	25	33	48	58	68	72	75	83	93	100	100
		35–44	201	36	40	50	61	72	75	79	86	97	100	100
		45–54	167	40	48	58	68	73	75	83	86	100	100	100
		55–64	136	38	40	56	68	75	75	83	86	93	100	100
		≥65	89	48	52	68	75	79	86	90	93	100	100	100
	At least one CHC	18–24	63	12	18	40	50	50	58	61	68	82	93	95
		25–34	125	8	16	33	47	54	61	70	75	84	97	100
		35–44	161	15	22	40	50	61	68	75	75	86	97	100
		45–54	173	12	25	43	54	61	68	72	79	86	98	100
		55–64	169	12	27	50	61	68	72	75	79	86	97	100
		≥65	124	36	40	50	61	68	72	79	83	93	100	100
Male	Healthy	18–24	117	33	39	50	58	65	72	75	79	86	97	100
		25–34	187	33	41	50	61	68	72	75	79	90	100	100
		35–44	242	25	43	50	68	72	75	75	83	93	100	100
		45–54	170	41	47	61	72	75	75	83	86	93	100	100
		55–64	134	50	53	65	75	75	79	86	90	97	100	100
		≥65	96	58	65	75	75	79	83	86	90	97	100	100
	At least one CHC	18–24	48	19	24	43	51	54	68	75	75	90	97	100
		25–34	94	11	22	36	47	50	54	58	65	76	87	96
		35–44	137	25	28	40	50	63	68	72	75	85	97	97
		45–54	154	21	25	43	54	61	68	72	75	86	97	100
		55–64	147	17	34	50	65	68	75	75	79	90	100	100
		≥65	123	25	33	54	68	72	75	79	83	92	100	100
Sex × Health Status × Education				Low HRQoL		−1 SD			MD			+1 SD	High HRQoL	
Sex	Health Status	Education	N	2.5%	5%	16%	30%	40%	50%	60%	70%	85%	95%	97.25%
Female	Healthy	Low	321	33	40	50	61	68	75	75	83	93	100	100
		Middle	445	33	40	54	66	74	75	83	90	97	100	100
		High	68	40	45	63	72	75	83	83	90	97	100	100
	At least one CHC	Low	296	11	18	40	50	58	68	72	75	86	97	100
		Middle	439	15	22	43	54	61	68	75	79	90	97	100
		High	80	18	36	50	58	68	72	79	83	90	100	100
Male	Healthy	Low	270	34	41	50	61	71	75	75	83	93	100	100
		Middle	576	40	50	58	68	75	75	79	83	93	100	100
		High	100	34	47	58	71	75	75	75	86	93	100	100
	At least one CHC	Low	224	22	33	47	54	65	68	75	75	86	96	98
		Middle	385	18	25	43	54	65	68	75	75	86	97	100
		High	94	25	31	40	50	58	65	68	75	90	100	100

Note: HRQoL: Health-Related Quality of Life; 50% percentiles represent 50% of the distribution corresponding to the median (*Md*); *SD*: Standard Deviation; values from −1 standard deviation (16%) to +1 standard deviation (85%) within the regular range (i.e., not impaired HRQoL). Values below 16% denote low HRQoL and values above 85% indicate outstanding HRQoL.

**Table A7:** Reference values for the QOLIBRI Social Relationships scale obtained from the general population sample in Italy stratified by sex, health status, age, and education.

Sex × Health Status × Age				Low HRQoL		−1 SD			Md			+1 SD	High HRQoL	
Sex	Health Status	Age	N	2.5%	5%	16%	30%	40%	50%	60%	70%	85%	95%	97.25%
Female	Healthy	18–24	82	21	38	50	60	67	71	75	75	88	100	100
		25–34	159	21	25	50	60	71	75	75	80	96	100	100
		35–44	201	30	30	50	63	67	75	75	80	96	100	100
		45–54	167	30	42	55	63	71	75	80	84	97	100	100
		55–64	136	30	33	53	67	71	75	75	80	88	100	100
		≥65	89	42	46	55	71	75	75	80	88	92	100	100
	At least one CHC	18–24	63	15	21	38	46	50	55	63	71	78	95	96
		25–34	125	9	14	38	50	55	63	67	75	84	95	96
		35–44	161	13	21	42	55	63	67	71	75	88	96	100
		45–54	173	9	17	34	50	59	67	71	75	88	98	100
		55–64	169	18	25	42	55	59	67	75	78	88	96	100
		≥65	124	26	34	46	59	64	71	75	80	88	96	100
Male	Healthy	18–24	117	21	30	50	55	63	67	75	75	84	97	100
		25–34	187	20	25	46	59	63	71	75	75	84	96	100
		35–44	242	26	38	50	59	67	75	75	80	92	100	100
		45–54	170	25	30	50	63	71	75	75	81	92	100	100
		55–64	134	27	37	56	67	75	75	80	84	96	100	100
		≥65	96	38	48	63	71	75	75	75	84	92	100	100
	At least one CHC	18–24	48	14	24	38	46	55	59	65	80	88	96	100
		25–34	94	13	13	33	42	47	50	58	67	75	92	95
		35–44	137	5	16	37	54	59	67	71	75	80	89	96
		45–54	154	9	13	36	46	55	65	71	75	84	96	100
		55–64	147	25	30	50	59	67	71	75	75	88	96	100
		≥65	123	22	34	50	63	67	71	75	84	91	96	96
Sex × Health Status × Education				Low HRQoL		−1 SD			MD			+1 SD	High HRQoL	
Sex	Health Status	Education	N	2.5%	5%	16%	30%	40%	50%	60%	70%	85%	95%	97.25%
Female	Healthy	Low	321	21	30	50	63	71	75	75	84	96	100	100
		Middle	445	26	34	50	63	71	75	75	80	96	100	100
		High	68	34	42	59	67	67	75	80	83	96	100	100
	At least one CHC	Low	296	13	17	42	53	59	67	71	75	88	96	100
		Middle	439	9	17	38	50	59	67	71	75	88	96	100
		High	80	25	38	46	55	59	65	71	75	80	92	92
Male	Healthy	Low	270	25	30	50	59	71	75	75	84	92	100	100
		Middle	576	25	34	50	63	67	75	75	80	88	100	100
		High	100	17	34	54	63	67	75	75	75	84	100	100
	At least one CHC	Low	224	13	25	45	55	63	71	75	76	88	96	100
		Middle	385	11	14	38	50	59	67	71	75	85	96	96
		High	94	14	21	37	50	51	59	66	75	88	98	100

Note: HRQoL: Health-Related Quality of Life; 50% percentiles represent 50% of the distribution corresponding to the median (*Md*); *SD*: Standard Deviation; values from −1 standard deviation (16%) to +1 standard deviation (85%) within the regular range (i.e., not impaired HRQoL). Values below 16% denote low HRQoL and values above 85% indicate outstanding HRQoL.

**Table A8:** Reference values for the QOLIBRI Emotions scale obtained from the general population sample in Italy stratified by sex, health status, age, and education.

Sex × Health Status × Age				Low HRQoL		−1 SD			Md			+1 SD	High HRQoL	
Sex	Health Status	Age	N	2.5%	5%	16%	30%	40%	50%	60%	70%	85%	95%	97.25%
Female	Healthy	18–24	82	6	10	25	35	42	50	50	60	75	95	100
		25–34	159	5	10	25	35	45	50	55	68	85	100	100
		35–44	201	15	20	30	40	50	50	60	75	85	100	100
		45–54	167	10	15	30	40	50	60	70	80	95	100	100
		55–64	136	25	25	35	50	55	65	75	85	95	100	100
		≥65	89	20	25	40	50	60	75	80	90	100	100	100
	At least one CHC	18–24	63	0	0	15	23	30	35	36	45	54	80	95
		25–34	125	0	2	20	25	30	35	45	50	65	85	90
		35–44	161	0	5	20	30	35	45	50	60	75	90	100
		45–54	173	0	5	25	34	40	45	55	65	81	95	100
		55–64	169	10	10	25	35	45	50	60	70	85	95	99
		≥65	124	15	16	30	45	55	65	74	80	90	100	100
Male	Healthy	18–24	117	10	10	25	35	45	45	50	60	78	95	100
		25–34	187	10	15	30	40	45	50	60	66	85	100	100
		35–44	242	20	25	30	45	50	60	70	75	90	100	100
		45–54	170	20	25	35	45	50	60	70	80	90	100	100
		55–64	134	17	25	35	50	65	70	79	90	100	100	100
		≥65	96	22	30	42	63	70	78	80	85	95	100	100
	At least one CHC	18–24	48	10	10	25	30	35	40	50	55	60	84	90
		25–34	94	0	10	25	35	40	50	50	55	65	84	99
		35–44	137	8	15	25	35	45	50	53	60	75	100	100
		45–54	154	10	15	25	35	45	50	55	65	85	100	100
		55–64	147	17	25	35	45	50	60	65	75	81	95	100
		≥65	123	20	21	40	50	55	65	72	80	90	100	100
Sex × Health Status × Education				Low HRQoL		−1 SD			Md			+1 SD	High HRQoL	
Sex	Health Status	Education	N	2.5%	5%	16%	30%	40%	50%	60%	70%	85%	95%	97.25%
Female	Healthy	Low	321	15	15	30	40	50	50	65	75	90	100	100
		Middle	445	10	15	30	40	50	55	65	75	90	100	100
		High	68	12	15	33	45	50	60	71	80	95	100	100
	At least one CHC	Low	296	0	0	20	30	40	45	50	65	80	95	100
		Middle	439	5	10	25	30	40	45	55	65	80	95	100
		High	80	10	15	25	35	44	50	55	65	80	85	96
Male	Healthy	Low	270	14	18	35	50	50	60	70	75	95	100	100
		Middle	576	15	20	30	45	50	60	70	80	90	100	100
		High	100	10	20	30	40	45	50	67	72	90	100	100
	At least one CHC	Low	224	10	16	30	40	50	50	60	70	83	100	100
		Middle	385	10	15	25	40	45	50	55	65	80	95	100
		High	94	5	14	30	40	40	50	55	60	75	90	94

Note: HRQoL: Health-Related Quality of Life; 50% percentiles represent 50% of the distribution corresponding to the median (*Md*); *SD*: Standard Deviation; values from −1 standard deviation (16%) to +1 standard deviation (85%) within the regular range (i.e., not impaired HRQoL). Values below 16% denote low HRQoL and values above 85% indicate outstanding HRQoL.

**Table A9:** Reference values for the QOLIBRI Physical Problems scale obtained from the general population sample in Italy stratified by sex, health status, age, and education.

Sex × Health Status × Age				Low HRQoL		−1 SD			Md			+1 SD	High HRQoL	
Sex	Health Status	Age	N	2.5%	5%	16%	30%	40%	50%	60%	70%	85%	95%	97.25%
Female	Healthy	18–24	82	20	26	45	50	52	65	70	75	90	100	100
		25–34	159	15	30	45	50	60	65	75	80	100	100	100
		35–44	201	25	25	45	50	60	70	75	85	95	100	100
		45–54	167	25	25	45	55	65	70	80	85	100	100	100
		55–64	136	22	29	40	58	65	75	80	85	90	100	100
		≥65	89	14	27	50	67	75	85	90	95	100	100	100
	At least one CHC	18–24	63	18	25	30	45	50	55	61	70	80	95	98
		25–34	125	5	15	25	40	50	50	60	70	80	94	100
		35–44	161	10	20	30	40	45	50	60	65	80	90	95
		45–54	173	5	10	30	40	45	50	62	70	80	98	100
		55–64	169	5	10	25	40	45	50	55	65	80	90	99
		≥65	124	20	25	34	45	52	60	69	80	85	90	95
Male	Healthy	18–24	117	15	25	40	50	55	65	70	75	88	95	100
		25–34	187	25	30	40	50	55	65	75	85	100	100	100
		35–44	242	25	30	45	55	60	70	75	85	100	100	100
		45–54	170	25	30	45	59	65	70	80	90	100	100	100
		55–64	134	30	40	52	70	75	80	80	90	100	100	100
		≥65	96	24	30	52	73	75	80	85	90	100	100	100
	At least one CHC	18–24	48	30	30	40	46	55	65	70	75	80	94	95
		25–34	94	20	24	40	50	50	60	65	75	80	100	100
		35–44	137	20	25	35	50	50	55	60	70	85	100	100
		45–54	154	10	15	35	45	50	55	65	70	80	92	100
		55–64	147	19	20	35	45	50	60	65	70	80	90	95
		≥65	123	16	20	40	50	55	60	65	75	85	90	95
Sex × Health Status × Education				Low HRQoL		−1 SD			Md			+1 SD	High HRQoL	
Sex	Health Status	Education	N	2.5%	5%	16%	30%	40%	50%	60%	70%	85%	95%	97.25%
Female	Healthy	Low	321	20	25	40	50	60	65	75	80	90	100	100
		Middle	445	15	25	45	55	65	75	80	85	100	100	100
		High	68	25	35	45	60	70	73	80	85	100	100	100
	At least one CHC	Low	296	5	10	30	40	50	50	60	70	80	95	100
		Middle	439	10	15	30	40	45	50	60	70	80	95	100
		High	80	10	20	35	44	50	60	65	70	80	86	91
Male	Healthy	Low	270	14	25	45	50	60	70	75	80	95	100	100
		Middle	576	25	30	45	55	65	75	80	85	100	100	100
		High	100	30	35	50	55	65	75	75	80	96	100	100
	At least one CHC	Low	224	10	20	35	50	50	55	65	70	85	95	100
		Middle	385	15	20	35	45	50	60	65	75	80	90	100
		High	94	25	25	40	45	50	55	60	70	85	95	95

Note: HRQoL: Health-Related Quality of Life; 50% percentiles represent 50% of the distribution corresponding to the median (*Md*); *SD*: Standard Deviation; values from −1 standard deviation (16%) to +1 standard deviation (85%) within the regular range (i.e., not impaired HRQoL). Values below 16% denote low HRQoL and values above 85% indicate outstanding HRQoL.
